# Effectiveness of Lymphedema Prevention Programs With Compression Garment After Lymphatic Node Dissection in Breast Cancer: A Randomized Controlled Clinical Trial

**DOI:** 10.3389/fresc.2021.727256

**Published:** 2021-11-26

**Authors:** Maria J. Nadal Castells, Eliot Ramirez Mirabal, Jordi Cuartero Archs, Jean C. Perrot Gonzalez, Marta Beranuy Rodriguez, Alberto Pintor Ojeda, Helena Bascuñana Ambros

**Affiliations:** Physical Medicine and Rehabilitation Department, Hospital de la Santa Creu i Sant Pau, Barcelona, Spain

**Keywords:** treatment, rehabilitation, prevention, breast cancer, lymphedema

## Abstract

**Background:** Patients with breast cancer who undergo axillary lymph node dissection (ALND) are at risk of developing lymphedema, which can negatively impact quality of life. Lymphedema prevention programs, which primarily consist of educational content and exercise, have been shown to reduce the incidence of lymphedema. The addition of compression garments (CG) may increase the effectiveness of these programs.

**Aim:** We aimed to determine whether adding a compression garment to a conventional lymphedema prevention program could improve treatment effectiveness.

**Design:** Randomized clinical trial.

**Methods:** Seventy patients who had undergone ALND for breast cancer were randomized to receive conventional preventative therapy (control arm, *n* = 35) consisting of a 1-hour educational session and a 12-week exercise program or the same therapy plus upper limb CGs (experimental arm, *n* = 35). Patients in the experimental arm were instructed to wear the CG ≥ 8 h/day for the first 3 months after surgery and 2 h/day thereafter.

**Results:** At 2-years, the overall incidence of lymphedema in the two groups was 12.3%, with no significant differences between the conventional and experimental arms (12.5 vs. 12.1%). In the experimental arm, the incidence of lymphedema was significantly lower (*p* = 0.02) in patients who used the CGs daily as recommended compared to patient who did not adhere to this treatment recommendation. Neither exercise (*p* = 0.518) nor education alone decreased the incidence of lymphedema. Adherence decreased over time.

**Conclusions:** The findings of this RCT show that health education, preventive exercise programs, and patient adherence to therapeutic recommendations all play an important role in preventing lymphedema.

**Clinical Rehabilitation Impact:** Our data demonstrated that the use of a compression garment during the first 3 months after axillary node dissection may reduce the likelihood of lymphedema in high-risk patients.

## Introduction

Breast cancer is the second most common cancer type worldwide. Improvements in diagnosis and treatment have substantially reduced the mortality rate in the last 50 years (1). However, patients who undergo axillary lymph node dissection (ALND) have an increased risk of developing lymphedema (2), with an estimated incidence rate of 21.4% (3).

Lymphedema is a chronic but treatable process with no definitive cure. This condition can cause significant physical and psychological disability, not only due to clinical alterations but also to the negative effects on work, home, and leisure activities, which can significantly decrease quality of life (QoL) (4). In addition to its impact on QoL, lymphedema has also been associated with a substantial increase in healthcare costs (5), which are significantly higher in patients with lymphedema (6). Importantly, studies show that the costs associated with prevention and early diagnosis programs are lower than those of treating lymphedema (7).

Although the importance of understanding the risks of lymphedema after treatment for breast cancer is widely recognized, patients frequently do not receive basic information about these risks (8). As a result, many patients are unaware of the risk factors and prevention strategies for lymphedema (9). For these reasons, health education program is essential in prevention of lymphedema.

The benefits of exercise in the treatment of lymphedema have been confirmed in numerous studies (10–13). In patients who develop lymphedema, the use of compression garments (CG) is considered a mainstay of therapy (14). However, the prophylactic use of these garments to prevent lymphedema has received relatively little attention. We have found randomized clinical trials that demonstrate the use of compression garments in the first 2 years after surgery reduces the incidence of lymphedema (15).

In this context, we performed a clinical trial to determine whether an experimental lymphedema prevention program consisting of exercise, health education, and the prophylactic use of a compression garment would be more effective than a conventional program alone in preventing lymphedema at 2 years. Evaluating the effectiveness of each components of this program is a secondary objective of this study.

## Patients and Methods

### Study Design

This was a single-centre, open-label, randomized controlled clinical trial. Patients were recruited from March 2011 to April 2013 from the Breast Pathology Unit at the Hospital de la Santa Creu i Sant Pau (HSCSP), a tertiary referral hospital in Barcelona.

### Study Participants

Inclusion criteria were as follows: age 18–85 years; having undergone ALND as part of treatment for primary breast cancer; and acceptance of study conditions and signed informed consent. Exclusion criteria were: presence of recurrent or metastatic cancer; open wounds or loss of skin integrity; dependency or deterioration of higher functions; arterial insufficiency; deep vein thrombosis; acute heart failure; severe peripheral neuropathy; and/or presence of lymphedema.

Participants were randomized to receive conventional preventive therapy or an experimental preventative program. The conventional prevention program consisted of a 1-h educational session and a 12-week exercise program. The experimental arm consisted of the same program plus the prophylactic use of CGs.

The conventional program, common to both groups, consisted of:

- A 1-h presentation by a trained physician and physiotherapist who reviewed the etiology and clinical manifestations of lymphedema, the risks of developing it, and recommended preventive measures. The recommended measures were based on those from the general consensus of the International Lymphedema Society, the Spanish Rehabilitation Society and The National Breast and Ovarian Cancer Centre (9) (Tables 1, 2).
- A 12-week in-person exercise program starting 7 days after ALND. Patients attended two weekly exercise sessions (60 minutes each) at the physiotherapy department for a total of 12 weeks. The program consistedof aerobic exercise combined with resistance and stretching exercises (see Annex 1). Patients were advised to continue with the exercises at home, both during the 12-week program and thereafter.

**Table 1:** Injury prevention measures.

**Injury prevention measures**
Use gloves for cleaning and for working in the garden Avoid animal scratches and mosquito bites Avoid burns Don't carry heavy weights; distribute weight between both hands Use an electric razor for hair removal, no waxing or razor blades Use a thimble when sewing Take good care of fingernails; do not cut cuticles Do not have blood pressure taken on the affected side Avoid blood draws on the affected limb Avoid injections on the affected side Do not have acupuncture Clean any wound with soap and water and apply an antiseptic Do not walk barefoot

**Table 2:** Hygiene and personal care measures.

**Hygiene and personal care measures**
Avoid extreme heat and cold Use sun protection Do not use irritating cosmetics Dry and hydrate the skin well after a shower or bath Do not wear a watch, bracelets or rings Do not wear clothing that is tight at chest, shoulder, waist or leg level Do not use saunas or UVA rays Avoid being overweight and weight gain; follow a low-fat diet Sleep on the non-operated side with the affected limb slightly elevated Consult your doctor in case of rashes or skin irritations Avoid vigorous and repetitive exercises Perform the set exercises daily Bandage the arm on flights longer than 2 h

The experimental group was also prescribed a flat knit, class 1 CG. Participants assigned to this group were instructed to wear the CG for 8 h during the daytime for the first 3 months after surgery and to remove it at night. Starting at month 4, the patients were instructed to wear the CG for 2 h per day, especially when performing exercises and physical activity.

The study conformed to the criteria established by the Declaration of Helsinki and the Guidelines for Good Clinical Practice. The study design was approved by the clinical research ethics committee at our institution (HSCSP). All patients signed the written informed consent form. The study protocol was registered at ClinicalTrials.gov (NCT04785599).

## Outcomes

All patients were evaluated at the start of the study (baseline), at the end of the 12-week exercise program, and thereafter at 6 months, 1 year, and 2 years. In all of these assessments, we measured the volume of the upper limbs using the lymphedema calculation formula based on the truncated cone, which has been validated and published by the Spanish Society of Physical Medicine and Rehabilitation (SERMEF) (16).

The presence of lymphedema was defined as a difference of >200 ml in volume between the upper extremities, or as a difference of 10% in volume between the two upper limbs (16).

We recorded all relevant parameters of the patients' medical history and general clinical examination, including TNM stage, treatments performed, number of resected nodes, and complications. Compliance with all the components of each program was monitored. Attendance at the educational session and at the 24 exercise sessions was recorded. Home compliance was monitored through personal interviews at the three follow-up assessments (at 6 months, 1 year, and 2 years). Patients who attended all three follow-up assessments were considered completers while those who missed any of these were considered non-completers.

## Statistical Analyses

Patients were randomized to the study groups using a table of random numbers generated with the IBM-SPSS statistical program, v.19. The health care staff involved in evaluating patient response to treatment were blinded to the treatment allocation.

The chi-square test was used to compare the two groups at baseline. To compare quantitative variables, Student's *t*-test for independent data was used; for ordinal variables, we used the non-parametric Mann-Whitney *U*-test. Analysis of variance (ANOVA) of repeated measures was used to study changes in the variables as a function of lymphedema over time. *P*-values ≤ 0.05 were considered significant. All statistical analyses were performed with the IBM-SPSS, v. 19.0 software program.

## Results

**Figure 1:**
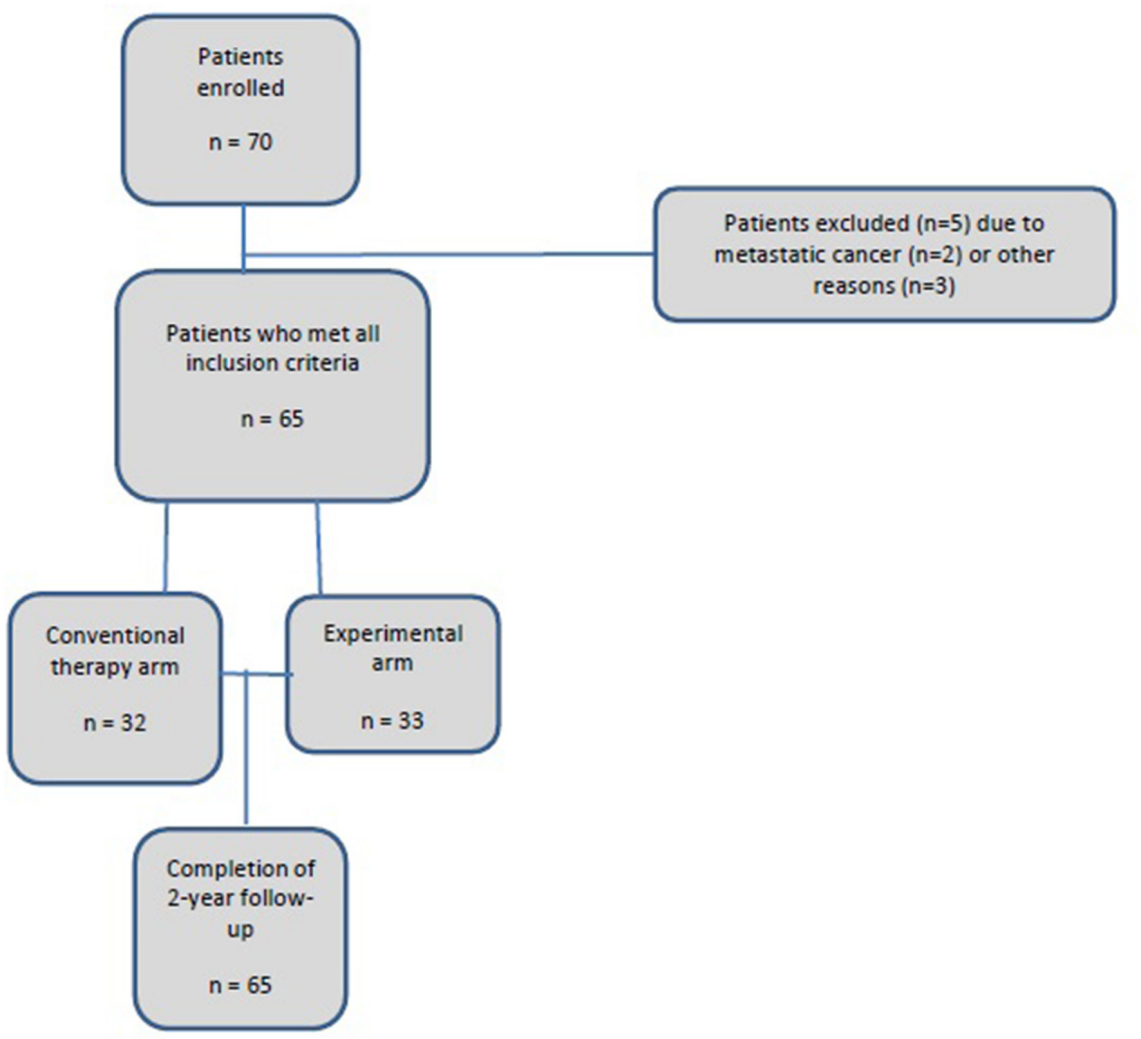
Study flowchart.

A total of 70 patients were enrolled in the study (35 in each arm). The mean age was 57.4 years (range, 26.5–82.8). Sixty-five participants (92.9%) completed the 2-year program. Five participants (7.1%) dropped out after the first evaluation (Figure 1, study flowchart).

Thus, the final analysis included 32 patients in the conventional prevention program and 33 in the experimental arm. The characteristics of the sample are shown in Table 3.

**Table 3:** Patient sociodemographic and clinical characteristics by group.

	**Conventional therapy arm,** ***N* = 35**	**Experimental arm,** ***N* = 35**	***p*-value**
	*n* (%)^*^	*n* (%)^*^	
Age (SD)	58.86 (12.7)	56.11 (12.7)	0.159
Women	35 (100.0)	35 (100.0)	
**Treatments**			
Lumpectomy	21 (60.0)	19 (54.2)	0.449
Mastectomy	14 (40.0)	16 (45.7)	1
Radiotherapy	29 (82.8)	29(82.8)	0.333
Chemotherapy	25 (71.4)	28 (80.0)	0.373
Hormone therapy	28 (80.0)	23 (65.7)	0.424
Immediate reconstruction	2 (14.3)	3 (18.7)	1
Mean number of resected nodes: 1–15	22 (62.9)	23 (65.7)	
Mean number of resected nodes: 16–30	13 (37.1)	12 (34.3)	0.018^(^*^)^
Involvement of dominant arm	17 (48.6)	23 (65.7)	0.330
Web syndrome	7 (20.0)	2 (5.7)	1
BMI (SD)	26.64 (4.0)	26.61(4.0)	0.042^(^*^)^
Educational level			0.786
Read/write only	4 (11.4)	10 (28.5)	
Primary	12 (34.3)	5 (14.3)	
Secondary	8 (22.8)	5 (14.3)	
University	11 (31.4)	15 (42.8)	

*SD, standard deviation; BMI, body mass index.*

* *All data given as n (%) unless otherwise indicated*.

**Figure 2:**
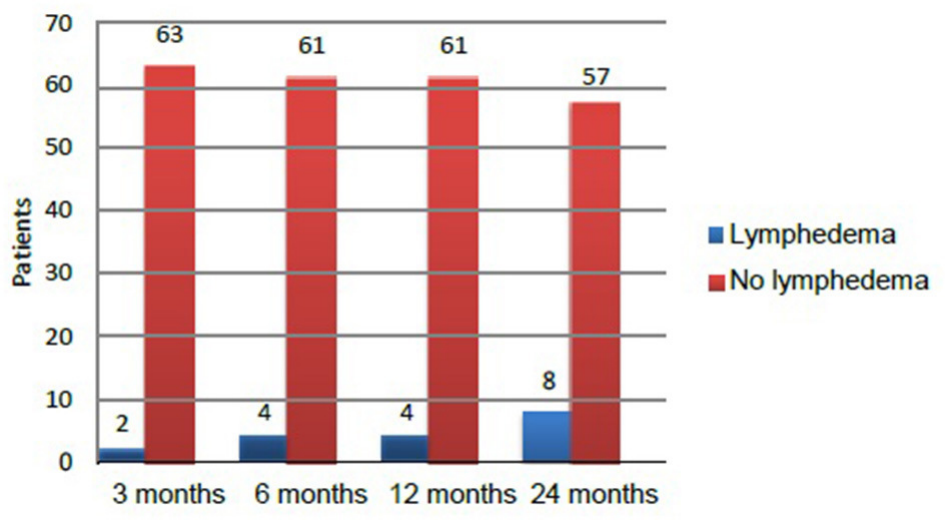
Time at which lymphedema developed during the 2 years follow-up in patients who completed the study (*n* = 65).

At the 2-year follow-up, the overall incidence of lymphedema in the 65 patients who completed the study was 12.3% (8/65). A total of eight patients (four in each arm) developed lymphedema, representing 12.5% of patients in the conventional arm vs. 12.1% in the experimental arm. No significant between-group differences were observed in the incidence of lymphedema. However, the incidence rate increased significantly (*p* = 0.028) over time for both (Figure 2).

### Prevention Program-Related Variables

Among the 65 patients who completed the 2-year assessment, there was no significant association (*p* = 0.518) between lymphedema at 2 years and adherence to the prescribed exercise regimen (Tables 4, 5).

**Table 4:** Lymphedema incidence at 2 years according to the frequency of exercises in the conventional prevention arm.

**Exercise**	**Presence of lymphedema**	**Absence of lymphedema**	**Total** **(*n* = 32)**
Daily	1 (12.5%)	7 (87.5%)	8
2–3 times/week	2 (13.3%)	13 (86.6%)	15
Occasional	1 (20%)	4 (80%)	5
None	0	4 (100%)	4

**Table 5:** Lymphedema incidence at 2 years according to the frequency of exercises in the experimental group.

**Exercise**	**Presence of lymphedema**	**Absence of lymphedema**	**Total** **(*n* = 33)**
Daily	2 (21.5%)	5 (71.5%)	7
2–3 times/week	1 (14.2%)	12 (85.7%)	14
Occasional	1 (14.3%)	6 (85.7%)	7
None	0	5 (100%)	5

In general, exercise adherence decreased over time. Patients who had previously exercised daily showed greater adherence to the prescribed exercise regimen and even an increased the amount of exercise after completing the in- person exercise program. These patients maintained this level for the first year.

**Figure 3:**
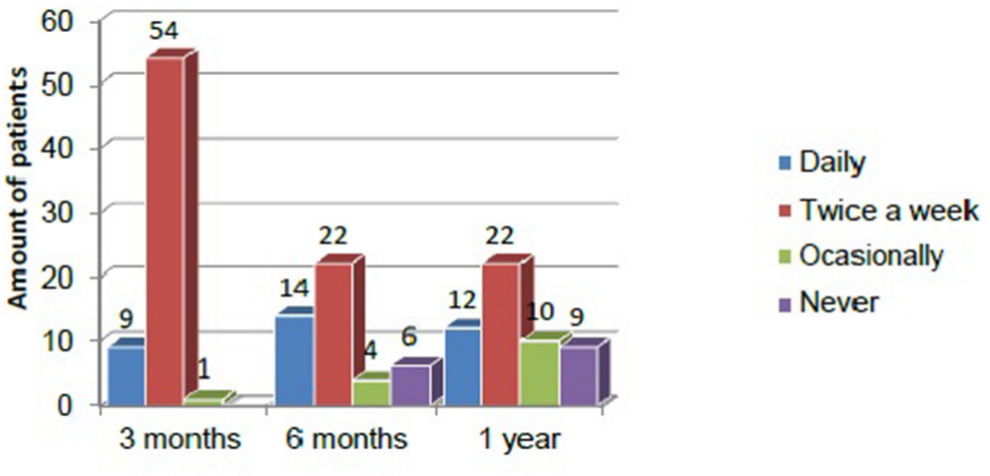
Adherence to exercise over the first year among completers (*n* = 60).

Among patients who exercised at least twice a week prior to study enrollment, the adherence rate during the in-person exercise program was 84.4%. However, after the 12-week exercise program ended, adherence decreased significantly (41.7% at 1 year). These results are shown in Figure 3.

Patients who used the GC daily during the first 3 months had a significantly lower incidence of lymphedema at 2 years than those who did use it daily (*p* = 0.020, Table 6).

**Table 6:** Lymphedema in the experimental arm as a function of frequency of CG use in the first 3 months.

**Frequency of compression garment use**	**Presence of lymphedema**	**Absence of lymphedema**	**Total** **(*n* = 33)**
Daily	1 (3.7%)	25 (96.2%)	26
2–3 times/week	1 (20.0%)	4 (80.0%)	5
Occasional	1 (100.0%)	0	1
Never	1 (100.0%)	0	1
Total	4 (12.1%)	29 (87.8%)	33

**Figure 4:**
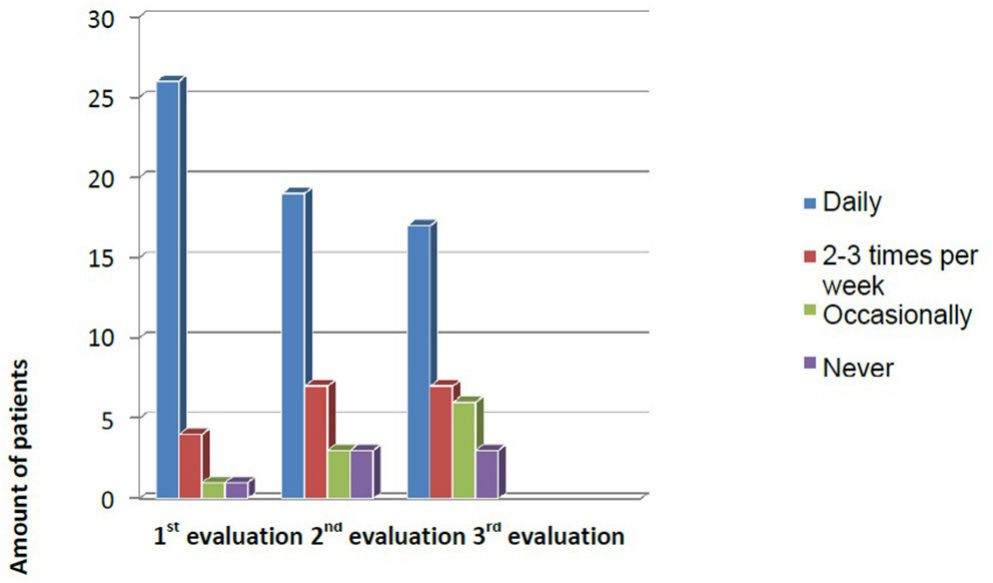
Evolution of adherence to CG use during the first year.

No significant association was found between adherence to the prescribed use of CG and lymphedema at 6 months, 1 year, or 2 years. Adherence decreased over time. At the end of the in-person exercise program, 78.1% of patients reported using the CG on a daily basis. However, at the 6-month and 2-year assessments, only 59.3 and 51.5%, respectively, continued daily use (Figure 4).

All of the patients enrolled in the study attended the educational session. In terms of compliance with the recommended hygiene and trauma prevention measures, all patients reported remembering the information received in the educational session and most (83.8%) affirmed that they had complied with all recommendations. However, the following recommendations were not met by all patients: use of gloves for cleaning and gardening (six patients did not follow this recommendation); no lifting weights (*n* = 4); use of a thimble when sewing (*n* = 3); fingernail care (*n* = 2); wound cleaning (*n* = 1); sleeping on non-involved side (*n* = 4); daily exercise (*n* = 24).

Statistical analyses of these variables showed no significant association between failure to comply with the recommended preventative measures and the subsequent development of lymphedema (data not shown).

## Discussion

This clinical trial was performed to determine whether the prophylactic use of compression garments combined with a conventional lymphedema prevention program would reduce the incidence of lymphedema at 2 years compared to conventional therapy alone. The overall incidence of lymphedema was low (12.3%), but without any significant differences between the conventional and experimental arms (12.5 vs. 12.1%). Importantly, however, in the experimental arm, patients who adhered to the recommended daily use of the CG during the first 3 months after surgery had a significantly lower incidence of lymphedema than those who did not (3.7 vs. 33.3%; *p* = 0.02). These findings are in line with previous reports (17, 18), confirming the effectiveness of prevention programs and supporting the use of compression garments after ALND.

In the systematic review carried out by Di Sipio et al. (3), the estimated incidence of lymphedema after breast cancer was 21.4%, but the incidence rate was highly variable. In most cases, the onset generally occurs within the first 2 years of treatment. Our results compare favorably with those described by Di Sipio and colleagues, with an incidence of only 12.3%.

Active exercise is one of the main components of lymphedema prevention programs. Exercising the limb at risk activates musculoskeletal pumping and increases venous and lymphatic flow. Upper body exercises can also restore the sympathetic impulse to the lymphatic vessels and thus help in the long-term management of lymphedema (19). Our exercise program was safe and we did not observe any association between exercise and the incidence of lymphedema. However, we were unable to demonstrate that daily exercise decreased the incidence of lymphedema in our patients, possibly due to the heterogeneity (intensity, type, adherence) of exercises performed at home and the difficulty of quantifying the amount of exercise performed in the home setting.

Our findings regarding the efficacy of exercise in preventing lymphedema are consistent with previous reports by Cavanaugh (7), Kwan et al. (10), and Cheema et al. (11), among others. Those studies also concluded that progressive exercise after ALND is safe and does not appear to promote the development of lymphedema. Baumann et al. (12) concluded that not only is strength exercise safe, but it may also have a preventive effect on the incidence of lymphedema in these patients.

The optimal time to start exercising after surgery is not clear. The studies conducted to date have varied widely in terms of both the timing and duration of the exercise program, which ranges from 4 weeks to 6 months. Importantly, to our knowledge, none of the studies performed to date have observed any significant association between resistance exercise and lymphedema, suggesting exercise can be safely initiated at any time after surgery for breast cancer.

Compression garments are the main treatment in patients who develop lymphedema. Compressive therapy reduces the formation of excess interstitial fluid, prevents lymphatic reflux, and helps muscle pumping by providing an inelastic barrier to the muscle (20). As a preventative measure for lymphedema, these garments could act through the same mechanisms. Our data appear to confirm the value of CGs as a preventative measure: in the experimental arm, patients who used a CG daily for 8 h in the first 3 months after surgery had a lower incidence of lymphedema at 2 years. By contrast, no preventive effect was observed in patients who used the CG only 2 h a day or only during exercise.

Although several RCTs have found that CGs can prevent lymphedema, there is only limited evidence to support this indication, mainly due to the limitations of those trials Castro-Sanchez et al. (21) conducted a clinical trial involving GCs combined with manual lymphatic drainage, finding that this combined therapy prevented lymphedema secondary to breast cancer surgery. However, the mean follow-up in that study was only 8 months. Stout et al. (22) evaluated the use of a CG worn during the entire day in the 4-week period immediately following surgery and thereafter only when performing strenuous activity. Crucially, none of the patients in that study developed lymphedema in the first year. In a clinical trial conducted by Ochalek et al. (15), the authors found that using a CG throughout the day for 3–12 months decreased the incidence of lymphedema.

The systematic review performed by Singh et al. (23) concluded that there was insufficient evidence to recommend the use of CG during exercise, which is consistent with our findings. In our study, CG was only preventive when worn for >8 h for the first few postoperative months. Our data suggest that wearing these garments does not appear to prevent lymphedema if used only during exercise. However, adherence to daily use of CGs is often poor, in part because wearing these garments in public is often poorly tolerated psychologically. In this regard, it would be useful to identify the characteristics of patients at high risk of developing lymphedema, which would allow for a more selective use of this preventive approach. However, this will require larger prospective studies with stricter patient selection.

Although most studies underscore the importance of complying with hygiene, personal care, and trauma prevention measures to prevent lymphedema, few have evaluated the efficacy of those recommendations. In our study, adherence to the recommended measures was close to 100%. However, due to the lack of a control group, we cannot demonstrate a significant association between adherence and the prevention of lymphedema. Nevertheless, our clinical experience strongly suggests that providing patients with relevant, health- related information is essential for prevention and that this strategy at least partially explains the low incidence of lymphedema in our sample in which both adherence and satisfaction rates were high. In fact, one of the main differences between our study and the other studies discussed here is that the majority of patients in those studies did not receive basic information about the risk of lymphedema after nodal resection. The role of other prevention variables, such as frequency of exercise and GC use, still need to be clarified.

In terms of modifiable risk factors, only one study (24) has demonstrated a clear association between lymphedema and body mass index (BMI). In that study, patients with a BMI > 25 kg/m^2^ had a 3-fold greater risk of developing lymphedema than patients with lower BMI values. Another study reported a higher incidence of lymphedema in patients who received chemotherapy to the involved limb (25).

## Conclusions

In this study, the incidence of lymphedema in both the control and experimental treatment arms was lower than reported in most studies. Crucially, the incidence of lymphedema was significantly lower in the patients who showed the highest level of adherence to the daily use of the compression garment during the first 3 months after axillary node dissection. Future studies are needed to determine whether this preventive approach could be adopted selectively in patients at high risk of *developing* lymphedema.

## Limitations

The main limitations of this study are the limited sample size and the difficulties associated with objective monitoring home-based exercise and CG use. In addition, although the 2-year follow-up was longer than in many studies, a longer follow-up would be useful to assess whether the low incidence rate observed is maintained over time. This would also allow us to objectively monitor use of CGs and the type, frequency, and intensity of exercise.

## Data Availability Statement

The original contributions presented in the study are included in the article/Supplementary Material, further inquiries can be directed to the corresponding author/s.

## Ethics Statement

The studies involving human participants were reviewed and approved by Institut de Recerca HSCSP (Santa Creu I Sant Pau Hospital Research institute) Cod: IIBS-EPC-2011-87. The patients/participants provided their written informed consent to participate in this study.

## Author Contributions

MN and HB: contributed to conception and design of the study and organized the database. MN: organized the database and performed the statistical analysis. ER, JP, and MB: wrote sections of the manuscript and data collection. JC: program execution and supplementary data. All authors contributed to the article and approved the submitted version.

## Conflict of Interest

The authors declare that the research was conducted in the absence of any commercial or financial relationships that could be construed as a potential conflict of interest.

## Publisher's Note

All claims expressed in this article are solely those of the authors and do not necessarily represent those of their affiliated organizations, or those of the publisher, the editors and the reviewers. Any product that may be evaluated in this article, or claim that may be made by its manufacturer, is not guaranteed or endorsed by the publisher.
