# Supplementary material for: Effectiveness of Lymphedema Prevention Programs With Compression Garment After Lymphatic Node Dissection in Breast Cancer: A Randomized Controlled Clinical Trial
Source: Front Rehabil Sci. 2021 Nov 26;2:727256. doi: 10.3389/fresc.2021.727256 (PMC9397767; doi:10.3389/fresc.2021.727256)

ANNEX1

Colegio de Fisioterapeutas de Cataluña. 20-3-21.
https://intranet.fisioterapeutes.cat/ca/fis/user_data/redirectEjerciciosTerapeutico

Wide breathing
stretching behind the head

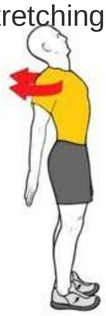

Posterior shoulder stretching

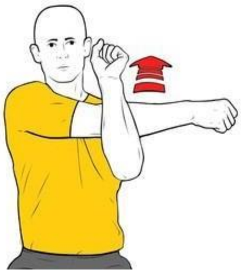

Triceps

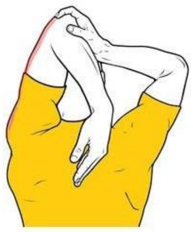

Upper trapezius stretching

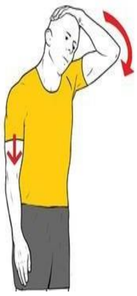

Shoulder stretching internal rotation

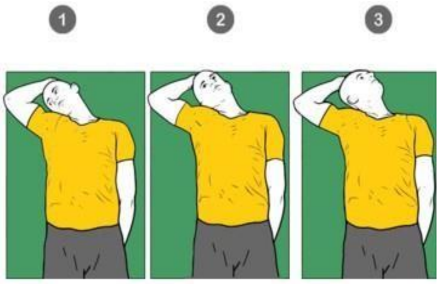

Stretching scalens

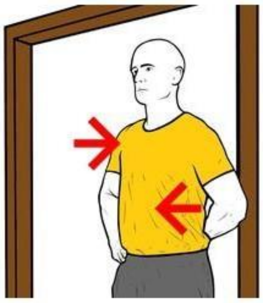

Stretching of trapeze and neck with arm on back

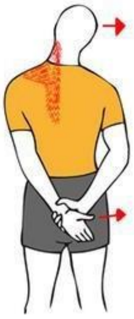

Pectoralis stretching with arm supported

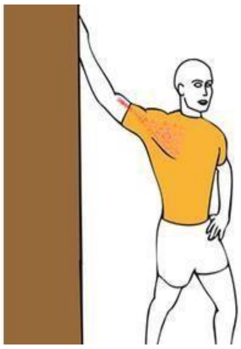

Elbow flexed pectoralis streching

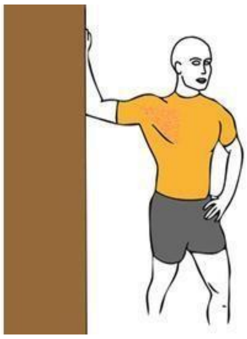

tching pectoralis and latissimus dorsi
with flexed trunk

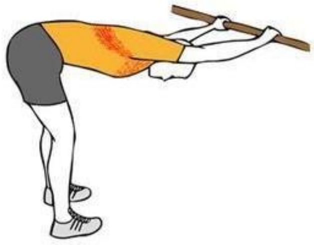

Waist twist with bar arms outstretched

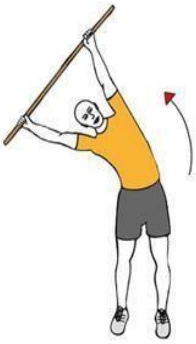

Stretching abdominal oblique and back

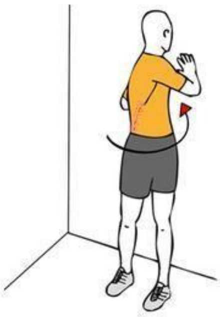

Stretching flexor carpi

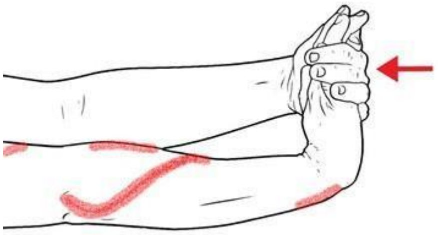

Lower trapezius activation

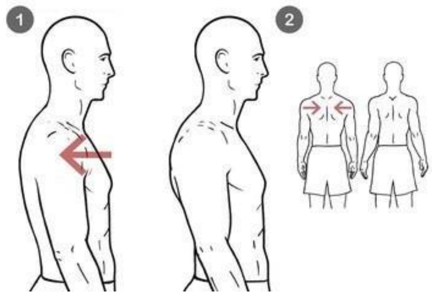

Stretching forearm muscles

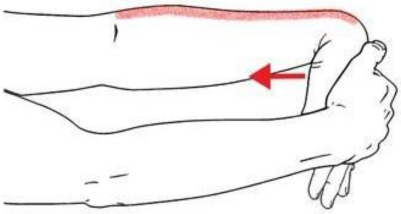

Control motor shoulder

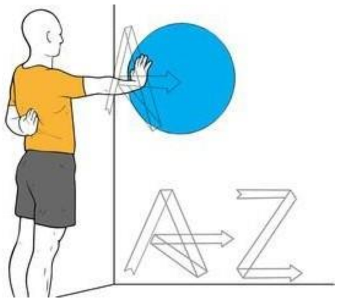

Lateral Control motor shoulder, capsular plane

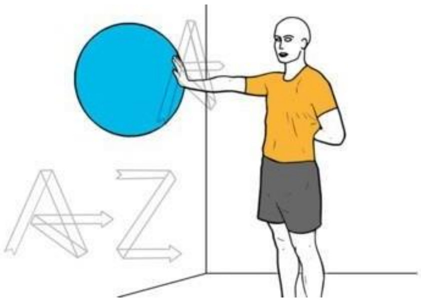

Birds with water bottles

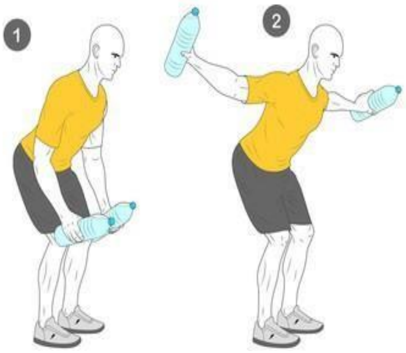

Front plank on the wall

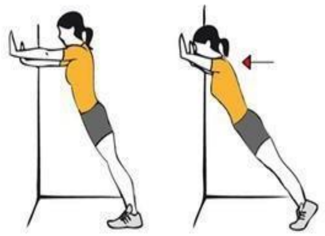

Stregthening rhomboideus

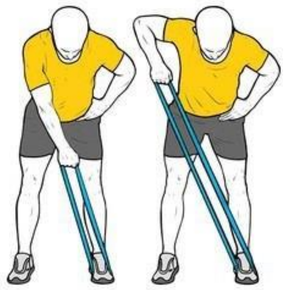

Triceps strengthening with elastic band

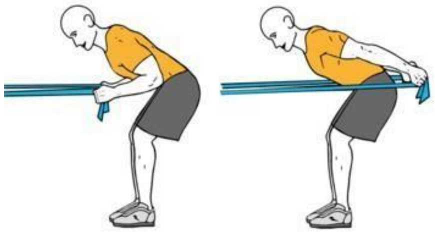

Triceps kicks with elastic band

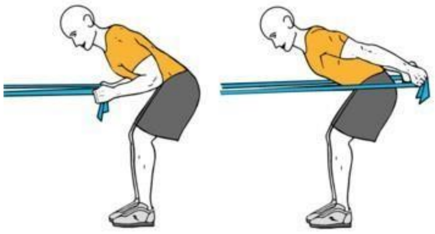

Elevation of shoulders in the plane of the scapula with elastic band

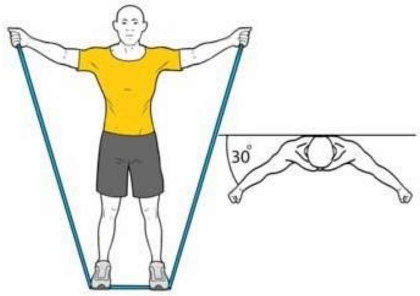

External rotation of shoulders with elastic band

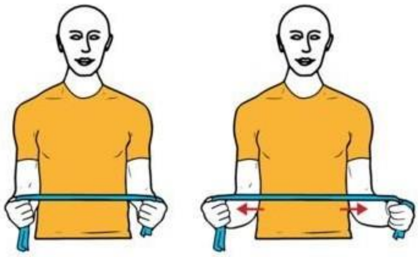

Supplement: Supplementary file 1 [file Data_Sheet_1.PDF]
